# Experimental quantification of useful and parasitic absorption of light in plasmon-enhanced thin silicon films for solar cells application

**DOI:** 10.1038/srep22481

**Published:** 2016-03-03

**Authors:** Seweryn Morawiec, Jakub Holovský, Manuel J. Mendes, Martin Müller, Kristina Ganzerová, Aliaksei Vetushka, Martin Ledinský, Francesco Priolo, Antonin Fejfar, Isodiana Crupi

**Affiliations:** 1 MATIS IMM-CNR, via S. Sofia 64, I-95123 Catania, Italy; 2 Dipartimento di Fisica e Astronomia, Università di Catania, via S. Sofia 64, I-95123 Catania, Italy; 3 Institute of Physics, Academy of Sciences of the Czech Republic, Cukrovarnicka 10, Prague, Czech Republic; 4 i3N/CENIMAT, Department of Materials Science, Faculty of Science and Technology, Universidade NOVA de Lisboa and CEMOP/UNINOVA, Campus de Caparica, 2829-516 Caparica, Portugal; 5 Scuola Superiore di Catania, Università di Catania, via Valdisavoia 9, 95123 Catania, Italy; 6 Department of Energy, Information Engineering and Mathematical Models (DEIM), University of Palermo, Viale delle Scienze, Building 9, I-90128 Palermo, ITALY

## Abstract

A combination of photocurrent and photothermal spectroscopic techniques is applied to experimentally quantify the useful and parasitic absorption of light in thin hydrogenated microcrystalline silicon (μc-Si:H) films incorporating optimized metal nanoparticle arrays, located at the rear surface, for improved light trapping via resonant plasmonic scattering. The photothermal technique accounts for the total absorptance and the photocurrent signal accounts only for the photons absorbed in the μc-Si:H layer (useful absorptance); therefore, the method allows for independent quantification of the useful and parasitic absorptance of the plasmonic (or any other) light trapping structure. We demonstrate that with a 0.9 μm thick absorber layer the optical losses related to the plasmonic light trapping in the whole structure are insignificant below 730 nm, above which they increase rapidly with increasing illumination wavelength. An average useful absorption of 43% and an average parasitic absorption of 19% over 400–1100 nm wavelength range is measured for μc-Si:H films deposited on optimized self-assembled Ag nanoparticles coupled with a flat mirror (plasmonic back reflector). For this sample, we demonstrate a significant broadband enhancement of the useful absorption resulting in the achievement of 91% of the maximum theoretical Lambertian limit of absorption.

Light trapping^1^ is an essential aspect in the design of solar cells based on thin absorbers, including both amorphous/microcrystalline thin films^2^,^3^,^4^ and the recently emerging thin mono-crystalline silicon technologies^5^,^6^, as it allows for the absorption of the long-wavelength (near-bandgap) photons due to the extended path-length of light inside the thin semiconductor. Among a broad range of approaches proposed to realize light trapping, the scattering of light from subwavelength metallic nanoparticles, due to the localized surface plasmon resonance (LSPR) effect, is often considered a promising route^7^,^8^, with a theoretical possibility to overcome the 4n^2^ limit^9^. In addition, the solid-state dewetting technique, commonly used for the fabrication of the metallic nanostructures^10^,^11^, gives additional advantages of low-cost, simplicity, direct scalability and compatibility with the industrial manufacturing processes. It has been demonstrated that such nanoparticles (NPs) incorporated in the so-called plasmonic back reflector (PBR) configuration – consisting of flat silver mirror, aluminum doped zinc oxide (AZO) spacer layer and the NPs – used as a substrate for the deposition of the photovoltaic absorber, can provide efficient light trapping comparable to state-of-art random texturing^12^,^13^.

In a solar cell structure, all the supporting layers and scattering elements additional to the photovoltaic material are sources of parasitic absorption, meaning that part of the incident sunlight absorbed by the device does not contribute to the obtainable photocurrent. The proper design of the device, and in particular the metallic nanostructures, is therefore an essential issue for the suppression of the optical losses. Valuable information on the distribution of absorption within the device can be provided by optical simulations using dielectric functions determined experimentally for each material^14^,^15^,^16^. However, for the optical response of self-assembled plasmonic NPs, notable discrepancies between computation and experiments have been observed^11^,^17^,^18^. This is often attributed to the presence of small particles in the nanostructures, the irregular shapes of the NPs, the inter-particle interactions, the sulfidation of NPs in atmospheric air, as well as to the polycrystalline nature, defects and impurities in the material forming the NPs^11^,^19^. In addition, particularly for plasmonic-based light trapping, the trade-off between the beneficial effects of scattering and the deteriorating effects of parasitic absorption can severely limit the overall photocurrent enhancement that can be produced in solar cells^20^,^21^,^22^.

Importantly, the contribution of useful and parasitic absorption cannot be measured separately with commonly used optical spectrophotometry. Therefore, in this paper we implement a combination of two absorption spectroscopy techniques, namely photothermal deflection spectroscopy (PDS)^23^ and Fourier-transform photocurrent spectroscopy (FTPS)^24^,^25^,^26^ in order to independently quantify the useful and parasitic absorption of light in plasmon-enhanced thin silicon films. The total absorption, contributed by the silicon, the silver NPs, all supporting layers and the substrate, is evaluated from the photothermal effect (PDS); while the fraction of light absorbed only in silicon (useful absorption) is measured based on the photoconductivity effect (FTPS). Although the proposed characterization method is not able to discriminate between the distinct sources of parasitic absorption – which is evaluated as the difference between the total and the useful absorption – it provides useful insights into the physical mechanisms of plasmonic light trapping as well as a first order prediction of the light trapping efficiency without the need to fabricate and process the entire device.

This method is used to determine the useful absorption enhancement in 0.9 μm thick films of hydrogenated microcrystalline silicon (μc-Si:H) provided by the distinct elements of the plasmonic back reflector configuration, at different stages of completion. It is demonstrated that the optical losses related to plasmonic light trapping in such structures are insignificant below 730 nm, beyond which they increase rapidly with increasing illumination wavelength. Furthermore, a significant broadband useful absorption enhancement of 90% is demonstrated, which resulted in achievement of 91% of the classical Lambertian limit of absorption. The improvements can be attributed to both the random front surface texture, originated from the conformal growth of Si on top of the NPs, and to the scattering of light by the plasmonic NPs.

## Experimental Details

### Sample preparation

**Figure 1:**
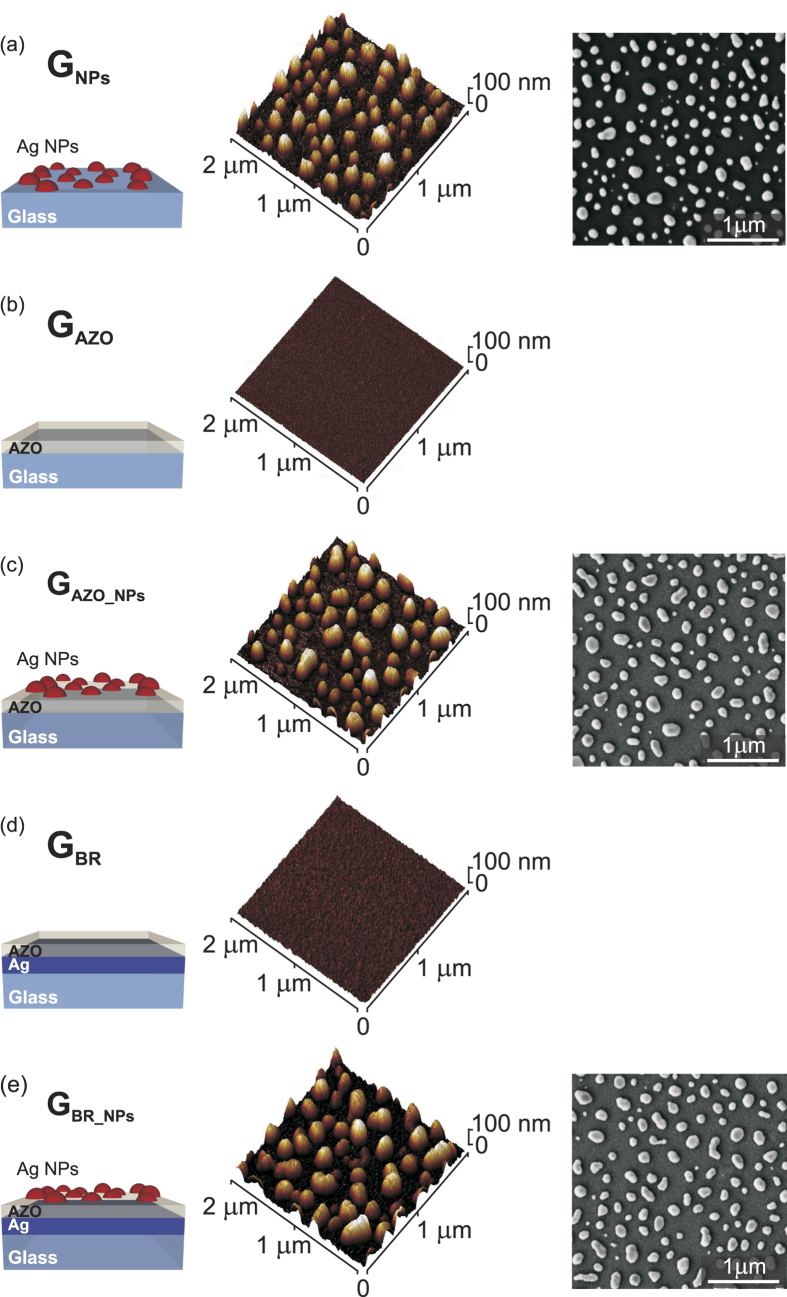
Schematic illustrations (*left*) and corresponding surface morphologies characterized by AFM (*center*) and SEM (*right*), of the five distinct substrate configurations used for the investigation of light trapping in μc-Si:H thin films deposited on top. G_NPs_ – Ag NPs on bare soda-lime glass; G_AZO_ – reference AZO-coated glass; G_AZO_NPs_ – NPs on AZO-coated glass; G_BR_ – reference Ag back reflector coated with AZO; G_BR_NPs_ – NPs on Ag back reflector coated with AZO (plasmonic back reflector). The NPs were fabricated in the same deposition process, from 12 nm thick Ag precursor films annealed at 400 °C for 1 h. The thicknesses of all AZO films is 50 nm.

In order to investigate the light trapping in substrate-configuration thin film solar cells, self-assembled silver nanoparticles were incorporated in three distinct arrangements, depicted schematically in Fig. 1(a,c,e), which are considered as different stages of completion of a plasmonic back reflector. The NPs were fabricated by solid-state dewetting (SSD) of 12 nm thick Ag films annealed at 400°C for 1 h in nitrogen atmosphere on: (i) bare soda-lime glass (sample G_NPs_), (ii) 50 nm thick aluminum doped zinc oxide (AZO) coated glass (sample G_AZO_NPs_), and (iii) a stack of 100 nm thick flat Ag back reflector (BR) and 50 nm thick AZO spacer layer (sample G_BR_NPs_). The reference samples without NPs (G_AZO_ and G_BR_, respectively) were fabricated in the same processes. The depositions of Ag and AZO films were carried out with RF magnetron sputtering at a working pressure of 2.5 × 10^−3^ mbar in Ar atmosphere with RF power density of 1 and 2.16 W/cm^2^, respectively. More details can be found in our previous works^11^,^20^.

The surface morphologies were investigated by Field Emission Scanning Electron Microscopy (SEM - Zeiss Supra 25 microscope) and Atomic Force Microscopy (AFM - Bruker Dimension Icon microscope in PeakForce mode). The optical properties of the NPs, in terms of total and diffuse reflectance (R_Total_ and R_Diff_, respectively), were measured using a Varian Cary 500 double-beam scanning UV-Vis-NIR spectrophotometer equipped with a 4-inch integrating sphere. A 0.9 μm thick μc-Si:H layer was deposited on top of the five structures shown in Fig. 1 by Plasma Enhanced Chemical Vapor Deposition (PECVD) with a power density of 0.06 W/cm^2^, a working pressure of 70 Pa, a SiH_4_ flow of 4 sccm, and a dilution ratio H_2_/SiH_4_ of 32, while keeping the samples’ surface at 310°C. The Si film thickness was verified with an Alpha-step 100 profilometer. The Raman spectra of the μc-Si:H films were acquired at 785 nm, with a low excitation intensity of 10 mW and accumulation time of 1 s, using a Renishaw InVia Raman spectrometer.

### Absorption spectroscopy

The absorption of light in the investigated structures was measured by highly sensitive photothermal deflection spectroscopy (PDS)^23^ and Fourier-transform photocurrent spectroscopy (FTPS)^24^,^25^,^26^. Both techniques exhibit superior sensitivity over transmittance/reflectance spectrophotometry and have been extensively used to analyze electronic defects in semiconductors.

**Figure 2:**
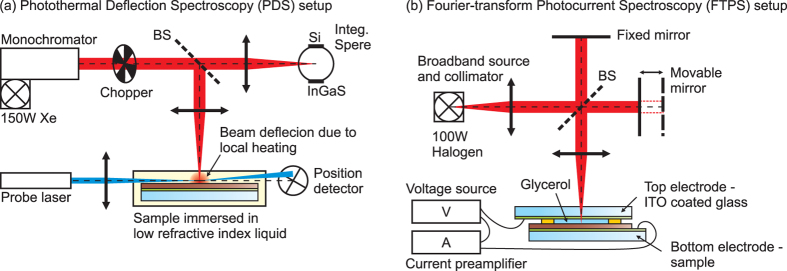
Schematic illustrations of the (**a**) photothermal deflection spectroscopy (PDS) and (**b**) Fourier-transform photocurrent spectroscopy (FTPS) setups. BS – beam splitter.

The PDS accounts for all absorption processes that result in the generation of heat. For the investigated samples (negligible luminescence quantum yield of μc-Si:H at room temperature) and measurement condition (no collection of photo-generated carriers), all photo-generated carriers thermalize and recombine non-radiatively generating heat. When the sample is immersed in liquid, the amount of generated heat can be measured precisely by the deflection of the laser beam caused by the local change of the refractive index of the surrounding liquid due to heating. Therefore, the PDS signal is proportional to the total absorption within the sample. The experimental setup used for PDS is depicted in Fig. 2(a). The sample is immersed in Fluorinert^TM^ Electronic Liquid FC-72 with a low refraction index of 1.25 to simulate conditions similar to ambient atmosphere. The sample is then illuminated with chopped monochromatic light from a monochromator with separate grating for UV, visible and IR coupled to a 150 W Xe lamp. A part of light is deflected by the beamsplitter into the integrating sphere equipped with Si and InGaAs photodiodes to monitor the light intensity. The probe beam from a He-Ne laser is directed parallel to the sample’s surface and focused in the heating spot. The amplitude of deflection is monitored by a position detector. The signal from the detector is coupled via the multiplexer to a current preamplifier and a lock-in amplifier referenced to the chopper frequency (13 Hz). The chopped illumination generates periodical thermal waves in the liquid surrounding the sample causing the periodical deflection of the laser beam. The amplitude of the deflection normalized to the reference black sample, such as a carbon nanotubes film on glass, gives the optical absorption spectrum of the investigated sample.

The FTPS signal is derived from the number of photo-carriers generated in the photovoltaic absorber and collected on external electrodes using applied bias. Therefore, it is proportional to the useful absorption, i.e. to the maximum photocurrent extractable from the photovoltaic material. The experimental setup used for FTPS is depicted in Fig. 2(b) and the theoretical principles of this method are described in detail elsewhere^24^. The setup is based on a Michelson interferometer and was built on a customized Fourier Transform Infrared (FTIR) Spectrometer (Thermo Nexus 8700) coupled with an external high intensity light source (100 W Halogen lamp) for better stability and higher signal. To collect an electrical signal from the sample, a specific arrangement was used comprising: (1) a top electrode made of a transparent conductive oxide window, (2) an electrolyte (glycerol) spacer, and (3) a sample with conductive AZO and/or silver mirror used as a bottom electrode. The voltage source and current preamplifier are connected in series with the electrodes. The interferogram is recorded in terms of current extracted from the sample. The use of Fourier-transform method is advantageous for the improved signal-to-noise (S/N) ratio by the high illumination intensity and high measurement speed that typically allows to collect and average few hundred of scans for one sample. FTPS can be understood as a standard FTIR method in which the investigated sample plays the role of a photodetector. Thus, the comparison with a calibrated detector having a known spectral response allows to determine the electrical response spectrum of the sample.

The FTPS method is valid generally provided that the photo-carriers are generated and collected homogeneously. The accuracy of FTPS is also affected by the band bending which is the limiting factor for the collection depth of the photo-carriers. Therefore, the method of signal collection becomes less accurate with increasing absorption coefficient, hence decreasing illumination wavelength, which causes inhomogeneous generation of carries and pronounced band bending. The accuracy of FTPS depends also on the sample thickness, conductivity of ohmic contacts and type of electrolyte. In our case of glycerol spacer and relatively thick sample, the relative error in the medium absorption range (600–800 nm) was estimated below 20%. The measurement conditions improve with increasing illumination wavelength providing improvements of the accuracy in the low absorption spectral region (800–1200 nm). As such, for our samples the FTPS was determined in the medium-low absorption range only.

The useful absorption of the Si film is equal to the FTPS absorption in the medium-low absorption range, while the total absorption is determined by the PDS. Therefore, the difference between the two measurements performed sequentially on the same sample determines the total parasitic absorption, which receives a contribution from all supporting layers and scattering elements. As such, the combination of PDS and FTPS spectroscopy allows for the independent quantification of the useful and total parasitic absorption, as attained by the conventional 1-R and EQE measurements usually performed in photovoltaic devices. The key advantage of the PDS + FTPS characterization approach is that it can be performed on the absorber layer alone, without requiring the full solar cell structure. Thus, it can be particularly interesting to evaluate the performance of light trapping structures coupled to the absorbers, without the interference of the additional elements (e.g. doped regions, contacts, window layers, etc.) required for the device completion.

## Results and Discussion

### Structural and optical characterization

The morphology of NPs fabricated in each configuration, analyzed by SEM and AFM, is depicted in Fig. 1. The average size of the NPs, determined from SEM images as the mean value of the Gaussian peak fitting to the distribution of NPs’ sizes, is found to be 139 ± 2 nm for glass and 169 ± 2 nm for the glass and AZO substrate. As a result of having the same underlying layer in the SSD process, the NPs’ morphologies of samples G_AZO_NPs_ and G_BR_NPs_ are fairly similar, though the NPs shapes were found to be slightly less uniform for the case of G_BR_NPs_. The AFM analyses reveal that the NPs have approximately hemispherical shape when formed on glass and tend to flatten out on AZO. The maximum heights were in both cases close to 100 nm. The SSD fabrication parameters employed in this work for the NPs formation resulted from thorough investigations performed by the authors aimed at optimizing the nanostructures’ optical properties^11^ and photocurrent enhancement that they produce in a-Si:H solar cells^20^.

**Figure 3:**
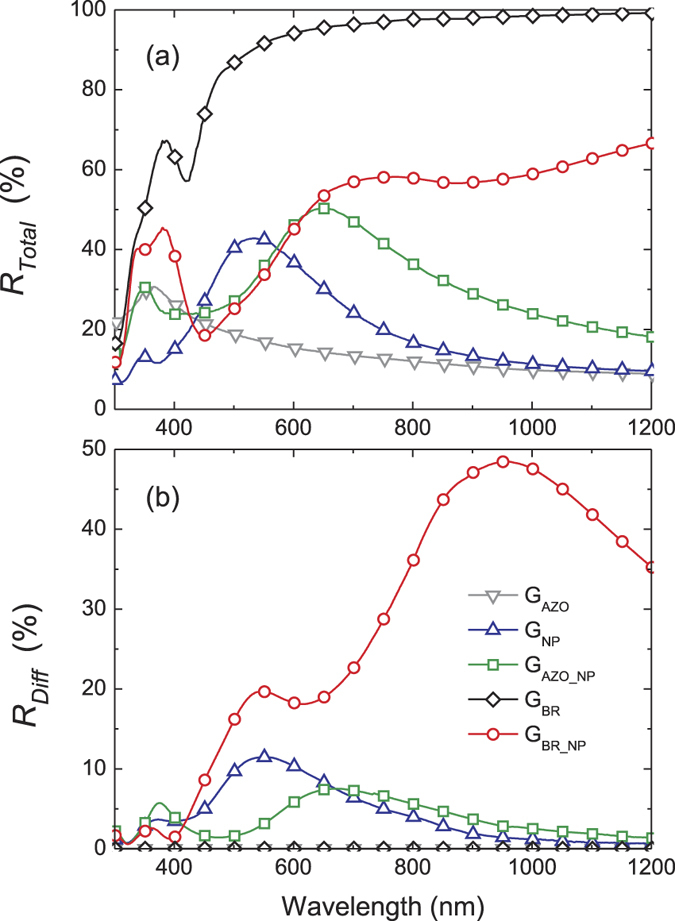
(**a**) Total and (**b**) diffuse reflectance of the five distinct substrate configurations depicted in Fig. 1.

Figure 3 depicts the optical properties, in terms of total and diffuse reflectance, measured in the front-side illumination configuration, of the five different substrates. An important parameter determining the optical performance of a sample for light trapping is the diffuse reflectance, as it corresponds to the amount of scattered photons which have an increased probability to be absorbed in the solar cell, relative to unscattered photons traversing the cell along the illumination direction. As expected, the two reference samples without NPs, G_AZO_ and G_BR_, show negligible R_Diff_. The NPs deposited on glass and AZO provide maximum diffuse reflectance of about 10 and 8% at 550 and 680 nm, respectively. The R_Diff_ is substantially enhanced in the wavelength range suitable for light trapping in μc-Si:H solar cells (500–1100 nm) when the NPs are coupled with a flat back reflector (sample G_BR_NPs_). This reveals that the mirror not only reflects the transmitted diffuse light coming from the NPs, but also originates a constructive interaction, which increases the intensity of the driving field of the NPs and therefore increases their scattered power relative to the case when they are immersed in a homogeneous medium^27^,^28^. Nevertheless, the dissipative interaction of the light with the NPs (parasitic absorption) is also substantially enhanced, resulting in the decrease of the total reflectance with respect to the R_Total_ of the reference mirror (sample G_BR_).

**Figure 4:**
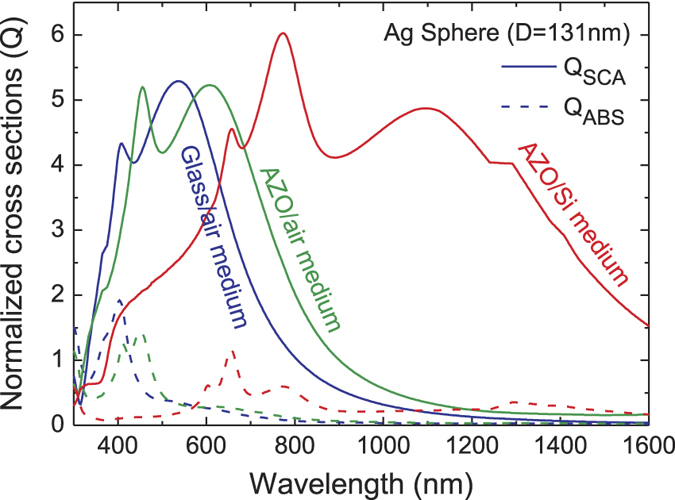
Calculated scattering (Q_SCA_) and absorption (Q_ABS_) cross sections, normalized by the physical area, of an Ag nanosphere embedded in different media. The particle diameter (D = 131 nm) is the average volume-equivalent sphere diameter of the Ag NPs fabricated in this work. The refractive index of each medium considered in the computations was taken as the average between that of the two materials surrounding the NPs in the structures of Fig. 1. These results were computed analytically with a Mie theory formalism^29^.

Figure 4 presents electromagnetic calculations of the normalized scattering (Q_SCA_) and absorption (Q_ABS_) cross sections obtained with a Mie theory formalism^29^. Such analytical method is based on a spherical particle surrounded by a homogeneous medium. Even though this condition is not satisfied in our samples, Mie theory can still be used for a first-order analytical prediction of the optical properties of Ag NPs embedded in different media, as performed in several previous studies related with plasmonic light trapping^7^,^28^,^29^,^30^,^31^. For the calculations in Fig. 4, we consider a single Ag nanosphere, with 131 nm diameter (the average volume-equivalent sphere diameter of the fabricated NPs) embedded in three different media with effective refractive indexes taken as the average between air and glass, air and AZO, and silicon and AZO. The spectral position and extension of the R_Diff_ peaks in Fig. 3, corresponding to G_NP_ and G_AZO_NP_, are similar to those of Q_SCA_ of a Ag NP in glass/air and AZO/air, respectively. As such, the inter-particle effects do not influence significantly the optical properties of the nanostructures. Furthermore, for such particle size, the parasitic absorption in the NPs arrays is expected to be small relative to their scattering effects as the Q_ABS_ peaks are much smaller compared to the Q_SCA_ ones. When the substrates of Fig. 1 are covered by the μc-Si:H thin films, the high refractive index of the Si medium causes the plasmonic modes of the Ag NPs to pronouncedly red-shift and broaden, as shown by the AZO/Si Q_SCA_ curve in Fig. 4. Therefore, when coupled to a Si layer, the three samples with NPs analyzed in this work should yield light scattering peaks overlapping with the preferential light trapping wavelength range (500–1100 nm) for thin film Si cells.

### Absorption enhancement in thin μc-silicon films

The five samples described in the previous section were used as substrates for the deposition of 0.9 μm thick μc-Si:H, with the aim of investigating plasmonic light trapping for substrate-configuration thin film Si solar cells. The selected deposition conditions assure high degree of crystallinity required for the narrow bandgap, which allow for the optical absorption to extend up to 1100 nm in the near-infrared. On the other hand, it was measured that the light is fully extinct in the film only for wavelengths below 500 nm. Thus, light trapping can provide absorption enhancement within the 500–1100 nm spectral window.

**Figure 5:**
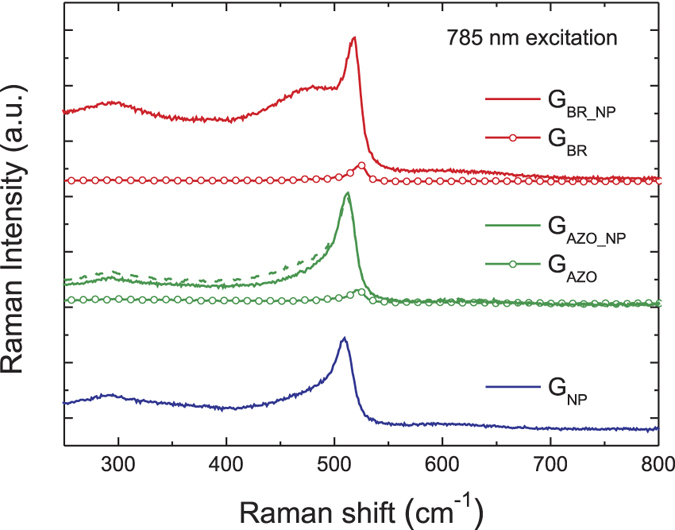
Raman spectra, measured with excitation at 785  nm, of 0.9 μm thick μc-Si:H deposited on the five different substrates depicted in Fig. 1. The peak at approximately 520 cm^−1^ originates from the microcrystalline phase of the films. The dashed curve refers to the sample G_AZO_NPs_ with an additional 50  nm thick AZO layer covering the NPs. The curves are displaced vertically in the graph for better visualization.

Figure 5 depicts the Raman spectra of μc-Si:H films deposited on the five different substrates. The high degree of crystallinity allows the clear identification of the Si band in the Raman spectra at approximately 520 cm^−1^. As discussed by Ledinský *et al.*^32^, the absolute intensity of the Raman signal is proportional to the path-length of the excitation light inside the silicon layer and to the in/out-coupling efficiency. Furthermore, the spectrum acquired for the configuration of sample G_AZO_NPs_, containing an additional 50 nm thick AZO layer covering the NPs (dashed line in Fig. 5) and separating them from the silicon layer, remains practically unchanged. This proves that surface enhanced Raman scattering (SERS)^33^, originated from the high near field enhancement in the NPs’ vicinity at the plasmon resonance, has a practically negligible impact on the measured increase of Raman intensity. Therefore, the observed enhancements of Raman signal for samples containing the NPs, over the flat Si film, can be attributed to the efficient far-field light trapping at the excitation wavelength; thereby providing an important insight into the enhancement of useful absorption in the Si material.

A crucial issue in plasmon-induced light trapping is to scrutinize between the absorption produced in the solar cell absorber layer (useful) and in the metallic NPs and supporting layers (parasitic), as the contributions of useful and parasitic absorption are inseparable with conventional optical spectrophotometry. To address that, an innovative procedure involving a combination of opto-electronic spectroscopic techniques, namely PDS and FTPS, was employed in this work. As described in the Experimental details, the PDS signal accounts for all types of light absorption which result in generation of heat, so it effectively measures the total absorption in the entire investigated structure. On the other hand, the FTPS absorption is determined from the number of photo-carriers generated in the photovoltaic absorber, thus accounting only for the useful absorption. Hence, the difference between the two measurements determines the total parasitic absorption.

**Figure 6:**
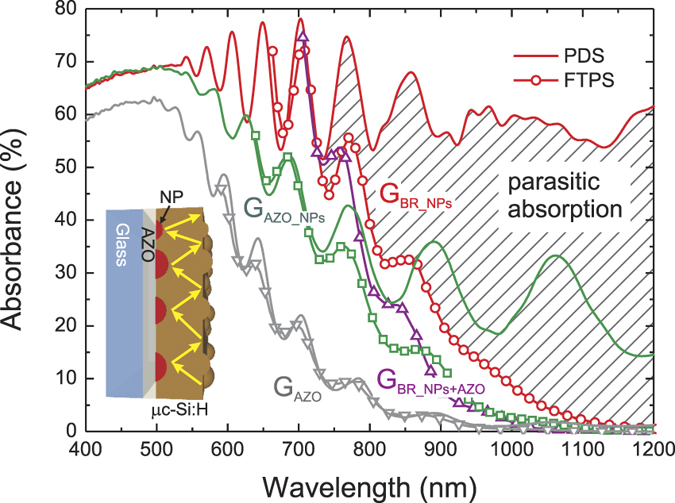
Total (PDS) and useful (FTPS) absorption spectra of 0.9 μm thick μc-Si:H films deposited on the samples G_AZO_, G_AZO_NPs_, and G_BR_NPs_ depicted in Fig. 1. The FTPS absorption for sample G_BR_NPs_, with an additional 50 nm thick AZO layer separating NPs and silicon (labeled G_BR_NPs+AZO_), is shown for comparison. The marked area is the difference between the PDS and FTPS spectra, which represents the parasitic absorption of sample G_BR_NPs_. The inset illustrates schematically the light trapped in the silicon layer by total internal reflection and scattering events on both interfaces, redirecting the light back to the silicon.

The PDS and FTPS absorption spectra of 0.9 μm thick μc-Si:H deposited on samples G_AZO_, G_AZO_NPs_, and G_BR_NPs_ are plotted in Fig. 6. In the reference G_AZO_ sample, both PDS and FTPS signals overlap, indicating that practically all absorption occurs in silicon and almost none in its AZO-coated glass substrate (only a small discrepancy, on the order of 1%, is apparent above 950 nm when plotting in logarithmic scale). It should be noted that the relatively low absolute values of absorption below 80% originate from the lack of the transparent contact deposited on top of silicon, which plays an important role of an antireflection coating.

The deposition of μc-Si:H on the substrates containing the NPs resulted in a considerable enhancement of the useful absorption in the investigated wavelength range. The parasitic losses, calculated as the difference between PDS and FTPS spectra, start to play a role only for wavelengths above 730 nm, which are poorly absorbed in flat Si films, and increase significantly towards the bandgap of μc-Si:H. The total parasitic absorption arises from multiple interactions between the light trapped in the silicon slab and the NPs, depicted schematically in the inset of Fig. 6. The number of such interactions for weakly absorbed near-infrared light increases substantially with decreasing absorption coefficient, hence increasing illumination wavelength. Consequently, the large number of interactions results in significant overall losses even though, as predicted by theoretical calculations^11^,^30^, the absorption cross-section of each single interaction is small compared to its scattering cross-section (shown in Fig. 4). As such, in the wavelength range below 730 nm, in which most light is absorbed in the first pass through the μc-Si:H (the 100% extinction of light occurs for wavelengths below 500 nm) and the remaining photons have high probability of being absorbed after only a few scattering events, the parasitic absorption plays only a minor role.

The main sources of parasitic losses can be attributed to the dissipative interactions of light with the NPs, the Ag mirror and AZO spacer layer. It should be noted that, even though no significant parasitic absorption is observed in Fig. 6 for the sample with only the AZO layer (G_AZO_), the losses in this layer or in the flat Ag mirror will necessarily increase with the presence of the nearby NPs. Although the exact values of the losses in each of these elements cannot be determined experimentally, they are all related to the plasmonic back reflector structure; thus, they should be accounted as the overall cost of the plasmonic light trapping implementation.

The deposition of μc-Si:H on self-assembled Ag NPs coupled with a flat mirror (sample G_BR_NPs_) resulted in an average useful absorption of 43% and an average parasitic absorption of 19%, calculated by integrating along the wavelength range of interest (400–1100 nm) for thin film Si solar cells. However, as observed in Fig. 6, the contribution of the parasitic absorption is only relevant for wavelengths above 730 nm for which the AM1.5G solar photon flux decreases markedly with increasing wavelength. Therefore, if we consider that our structure is a solar cell with unitary internal quantum efficiency, the attainable short-circuit current density (J_sc_) calculated from useful absorption (FTPS signal) would be 19.1 mA/cm^2^. This is 95% higher than that of the reference sample G_AZO_ without PBR (J_sc_ = 9.8 mA/cm^2^) and close to the maximum theoretical current of approximately 21 mA/cm^2^ that would be achieved for perfect Lambertian light diffusion on both front and rear interface^34^. The limit was calculated using dielectric functions of μc-Si:H form Jun *et al.*^35^ without antireflection coating. The significant broadband enhancement of the useful absorption achieved for sample G_BR_NPs_ resulted in the achievement of 91% of the classical Lambertian limit of absorption.

The scattering properties of particles are strongly dependent on the dielectric function of the embedding medium^30^, therefore also on the distance between the NPs and high refractive index material^31^,^36^. The FTPS signal of sample G_BR_NPs_ with an additional 50 nm thick AZO layer separating NPs and silicon (curve labeled G_BR_NPs+AZO_ in Fig. 6), exhibits a significant blue-shift of the absorption edge and thus a clearly lower enhancement of useful absorption in the near infrared region. This can be attributed to the decrease of the overlap between the NPs’ near field and the silicon, which results in lower absorption induced by the near field as well as the narrowing of the angular distribution function of scattered light, hence lower light coupling efficiency^31^,^36^. As such, from the optical point of view, the NPs-Si separation should be kept as thin as possible. Nonetheless, in a complete solar cell structure the spacer layer is required as a barrier for the diffusion of metal atoms into the silicon, thus preventing the deterioration of the electrical properties of the doped layer and degradation of the p-i-n junction. In addition, a sufficiently thick spacer is also required to prevent strong absorption in the n-type layer of the cell originating from the NPs’ near-field, which would result in high parasitic losses caused by the significant number of defects and low carrier mobility in the doped layers. This sets up a limit for the minimum thickness of the spacer layer, as discussed in detail by Tan *et al.*^37^.

The conformal growth of μc-Si:H films on top of the NPs results in a random texturing of their front surface. The surface morphologies were found to be fairly similar for the three films deposited on substrates containing NPs (G_NPs_, G_AZO_NPs_, and G_BR_NPs_), having root mean square (RMS) roughness ranging from 20 to 24 nm. As such, distinct light trapping mechanisms are contributing to the overall useful absorption and Raman enhancements: (i) antireflection action and surface scattering provided by the surface texture, and (ii) scattering of light by the plasmonic nanoparticles. The high energy photons (<500 nm wavelength) are entirely absorbed in their first pass through the μc-Si:H film and therefore do not reach the back side of the film and have no possibility to interact with the NPs. Therefore, the higher absorption measured at wavelengths shorter than 500 nm for samples with NPs can only be attributed to the front surface texture. In the light trapping window (500–1100 nm), both mechanisms can originate the observed enhancement of useful absorption and their contributions cannot be directly discriminated. Nonetheless, the computational study by Kowalczewski *et al.*^38^ shows that small RMS surface roughness affects predominantly the high energy photons, and its antireflection/scattering effectiveness decreases with increasing illumination wavelength. On the other hand, plasmonic scattering has impact at longer wavelengths, in the red and near-infrared part of the spectrum, due to the pronounced red-shift of the NPs resonance when placed in the vicinity of a high refractive index material^12^,^13^,^20^.

Although the two light trapping mechanisms are inseparable by the spectroscopic techniques used in this work, two main conclusions can be drawn. First, the significant difference of R_Diff_ between G_AZO_NPs_ and G_BR_NPs_ (see Fig. 3) indicates that the enhancement of useful absorption provided by the G_AZO_NPs_ sample should predominantly result from the textured front surface. Second, as the antireflection action provided by the surface texture is expected to contribute equally to light trapping in all samples containing NPs, and since the plasmonic scattering increases significantly in presence of a flat back mirror^27^,^28^, the increase of useful absorption between samples G_AZO_NPs_ and G_BR_NPs_ should mainly originate from the plasmonic light trapping. The latter claim is also supported by the blue-shift of the useful absorption edge observed for sample G_BR_NPs_ with a 50 nm thick AZO layer separating NPs and silicon.

**Figure 7:**
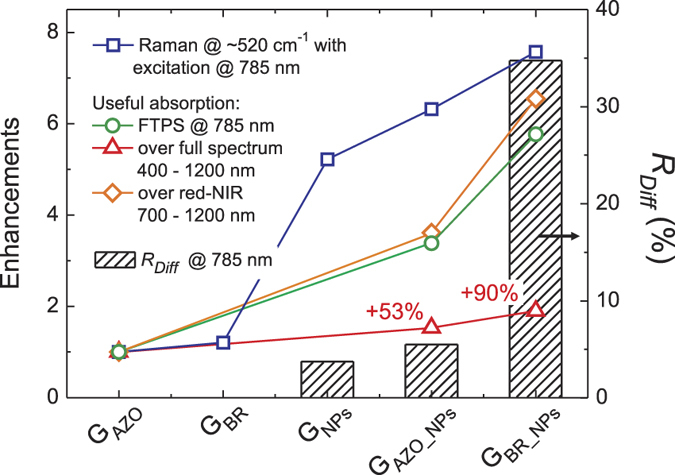
Enhancements (*left axis*) relative to the reference flat film (G_AZO_) of the Raman Si-band peak at ~520 cm^−1^, the FTPS useful absorption at 785 nm, the overall useful absorption integrated over 400–1200 nm, and the red-NIR part of useful absorption integrated over 700–1200 nm, provided by the different substrates depicted in Fig. 1. The enhancements are correlated with the diffuse reflectance (*right axis*) at 785 nm measured prior to the deposition of μc-Si:H.

Figure 7 depicts the enhancements in selected quantities, provided by the different substrates, over the reference flat film (sample G_AZO_). The FTPS (useful absorption) enhancements are compared to the diffuse reflectance at 785 nm, measured prior to the deposition of μc-Si:H films, which corresponds to the laser excitation wavelength used for Raman spectroscopy. The flat back reflector (sample G_BR_) can effectively double the path-length of light inside the flat μc-Si:H film, which is meaningful only in the range 500–700 nm where specularly reflected light has still a high probability of being absorbed in the second pass through Si. Thus, the Ag mirror of sample G_BR_ has been found to provide minor, 1.25, enhancement of the Raman signal acquired at 785 nm. On the other hand, much higher Raman enhancements of 6.3 and 7.6 were found for the films deposited on substrates G_AZO_NPs_ and G_BR_NPs_, respectively, due to the redirection of scattered light to more horizontal paths inside the films.

The deposition of μc-Si:H on self-assembled Ag NPs coupled with a flat mirror (sample G_BR_NPs_) resulted in a pronounced 90% enhancement of the useful absorption, in the entire investigated spectral range (triangular symbols in Fig. 7). Importantly, the enhancements of the red-NIR part of useful absorption and of the FTPS signal at 785 nm correlate reasonably well with the diffuse reflectance provided by the substrates containing NPs. Therefore, the results demonstrate that: (1) the experimentally-measured absorption enhancements occurring in the Si, in the red-NIR range, originated in fact from the light scattering caused by the NPs; and (2) the measurements of R_Diff_ can serve as a first approximation of the light trapping performance, while the combined PDS and FTPS spectroscopy method can be applied to obtain the refined quantities of absorption.

## Conclusions

A novel procedure employed in this work, involving a combination of opto-electronic spectroscopic techniques, namely PDS and FTPS, allowed for the quantification of useful and parasitic absorption in 0.9 μm thick μc-Si:H deposited on a plasmonic back reflector. It has been found that the optical losses related to the plasmonic light trapping for such structure are insignificant in the wavelength range below 730 nm, beyond which they increase rapidly with increasing illumination wavelength. This is explained by the substantial increase of the number of interactions between the NPs and the long-wavelength photons, due to the multiple internal reflections of light inside the Si film as a consequence of the rapid drop of the semiconductor’s absorption coefficient, which accounts for the overall losses. Nonetheless, a significant broadband useful absorption enhancement of +90% has been demonstrated, which results in achievement of 91% of the classical Lambertian limit of absorption. The improvements can be attributed to both the random front surface texture, originated from the conformal growth of Si on top of the NPs, and to the scattering of light by the plasmonic NPs. In addition, the experiment gives new insights into the field of light trapping and associated characterization tools; proving that optical R_Diff_ measurements are a reasonable first order approximation of the performance of scattering structures, while combined PDS and FTPS spectroscopy can be a more refined optoelectronic prediction of their light trapping efficiency when applied to actual devices such as thin film solar cells.

## Additional Information

**How to cite this article**: Morawiec, S. *et al.* Experimental quantification of useful and parasitic absorption of light in plasmon-enhanced thin silicon films for solar cells application. *Sci. Rep.* **6**, 22481; doi: 10.1038/srep22481 (2016).
